# Concomitant Light-Reversible Magnetic Response in Multiferroic Oxide Heterostructures for Multiphysics Applications

**DOI:** 10.1021/acsami.4c02551

**Published:** 2024-04-08

**Authors:** Jesús López-Sánchez, Adolfo Del Campo, Adrián Quesada, Alejandro Rivelles, Manuel Abuín, Raquel Sainz, Eugenia Sebastiani-Tofano, Juan Rubio-Zuazo, Diego A. Ochoa, José F. Fernández, José E. García, Fernando Rubio-Marcos

**Affiliations:** † Department of Electroceramics, Instituto de Cerámica y Vidrio—Consejo Superior de Investigaciones Científicas (ICV—CSIC), 28049 Madrid, Spain; ‡ Instituto de Sistemas Optoelectrónicos y Microtecnología (ISOM), Universidad Politécnica de Madrid (UPM), 28040 Madrid, Spain; § Instituto de Catálisis y Petroleoquímica—Consejo Superior de Investigaciones Científicas, (ICP—CSIC), 28049 Madrid, Spain; ∥ Instituto de Ciencia de Materiales de Madrid—Consejo Superior de Investigaciones Científicas (ICMM—CSIC), 28049 Madrid, Spain; ⊥ Spanish CRG BM25—SpLine at the ESRF—The European Synchrotron, 38000 Grenoble, France; # Department of Physics, Universitat Politècnica de Catalunya (UPC), 08034 Barcelona, Spain

**Keywords:** multiferroics, epitaxial thin films, magneto-structural coupling, photostrictive materials, charge distribution modulation, ferroelectric domain wall commutation

## Abstract

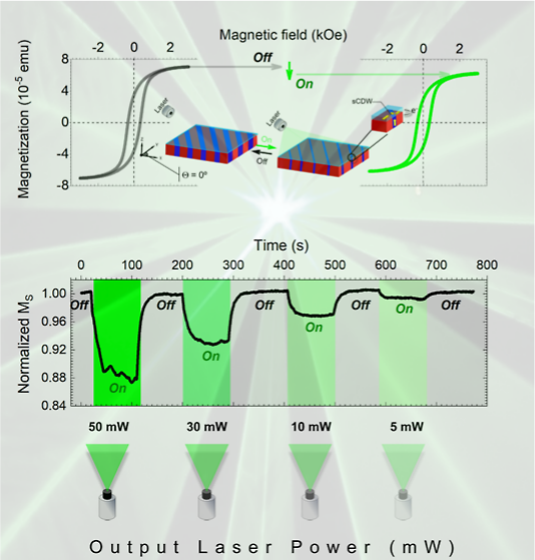

The concept of multiphysics, where materials respond to diverse external stimuli, such as magnetic fields, electric fields, light irradiation, stress, heat, and chemical reactions, plays a fundamental role in the development of innovative devices. Nanomanufacturing, especially in low-dimensional systems, enhances the synergistic interactions taking place on the nanoscale. Light–matter interaction, rather than electric fields, holds great promise for achieving low-power, wireless control over magnetism, solving two major technological problems: the feasibility of electrical contacts at smaller scales and the undesired heating of the devices. Here, we shed light on the remarkable reversible modulation of magnetism using visible light in epitaxial Fe_3_O_4_/BaTiO_3_ heterostructure. This achievement is underpinned by the convergence of two distinct mechanisms. First, the magnetoelastic effect, triggered by ferroelectric domain switching, induces a proportional change in coercivity and remanence upon laser illumination. Second, light–matter interaction induces charged ferroelectric domain walls’ electrostatic decompensations, acting intimately on the magnetization of the epitaxial Fe_3_O_4_ film by magnetoelectric coupling. Crucially, our experimental results vividly illustrate the capability to manipulate magnetic properties using visible light. This concomitant mechanism provides a promising avenue for low-intensity visible-light manipulation of magnetism, offering potential applications in multiferroic devices.

## 1 Introduction

In the ongoing pursuit of cutting-edge technological advances, the focus centers on the intersection of diverse physical phenomena (that is, multiphysics phenomena) and their versatile application. The concept of multiphysics, understood as materials that react to a spectrum of external stimuli, yielding an array of physicochemical responses, has become a cornerstone within the realm of materials science. The range of stimuli encompasses magnetic and electric fields, light irradiation, mechanical stress, heat, and chemical reactions, unveiling a set of functionalities in the same material.^1−4^ This trajectory finds its embodiment in devices characterized by their simplicity and cost-effectiveness, within which the photostimulation offers immediate and accurate responses in a controllable fashion. Photostimulation’s unique capacity to provide monitorable responses sans circuitry or substantial thermal impacts positions it at the forefront of industrial and technological innovation, boosting the development of light-driven devices across various fields.^5−14^

An interesting avenue within this versatile scenario emerges in the framework of multiferroic heterostructures. These materials inherently accommodate simultaneous electric and magnetic orders in specific temperature ranges, offering a complex interplay of multiphysics behaviors.^4,15^ As research surges forward in fields like transistor-based random-access memories and spintronics, the intricate interactions of magnetoelastic and magnetoelectric couplings in ferromagnetic–ferroelectric metal structures have taken center stage.^16−18^ More specifically, a promising trend materializes through the emergence of multiferroic heterostructures based on ferroelectric–ferromagnetic oxides, showcasing potential for enhanced stability and tunable properties governed by localized electrons.^19,20^ However, these studies often rely on electric currents for tailored responses, facing challenges encompassing irreversible transitions, Joule-induced overheating, dielectric degradation, and vulnerabilities in tunnel barriers.^17,21−24^

In this investigation, we embark on an exploration centered around the multiferroic Fe_3_O_4_/BaTiO_3_(100) system. Magnetite (Fe_3_O_4_), renowned for its well-known ferromagnetism, magnetostriction, and half-metallic attributes,^20,25^ is known to grow epitaxially on BaTiO_3_, a quintessential archetype of ferroelectric perovskite materials.^20,26^ This harmonious fusion, based on the crystalline coherence at the interface between both materials, engenders a versatile spectrum of functionalities, spanning from electrochemical reduction and spintronics to the intricacies of multiferroicity.^20,25−32^ In particular, previous studies have shown that, as a consequence of magnetoelectric coupling, the magnetic properties of Fe_3_O_4_ can be indirectly affected by temperature and electric fields that change the ferroelectric order and/or strain state in BaTiO_3_.^33−36^ Here, we report for the first time a completely reversible modulation of the magnetization of Fe_3_O_4_ by means of low-intensity visible light illumination, a consequence of the magnetoelectric coupling with the light-induced ferroelectric domain rearrangement within BaTiO_3_.

## 2 Materials, Methods, and Experimental Details

### 2.1 Fabrication of Fe_3_O_4_/BaTiO_3_ Heterostructure by Pulsed Laser Deposition

**Figure 1:**
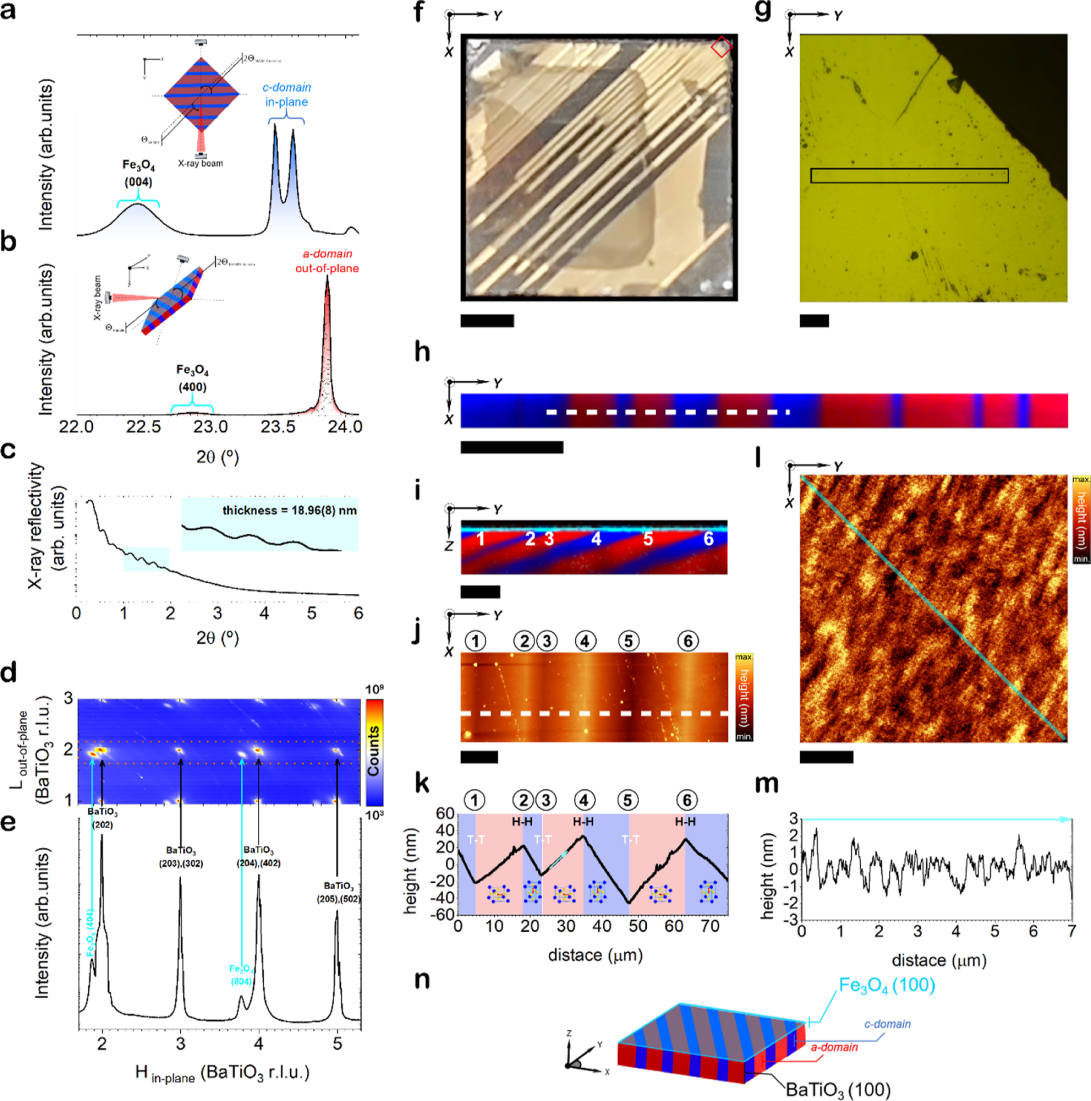
Exhaustive characterization of the multiferroic Fe_3_O_4_/BaTiO_3_ heterostructure. (a) High-resolution in-plane and (b) out-of-plane X-ray diffraction (XRD) patterns. The observed BaTiO_3_ and Fe_3_O_4_ reflections are indicated in each case, respectively. Note that due to the high photon flux delivered by synchrotron light, pure in-plane and out-of-plane components can be completely decomposed enabling the unambiguous visualization of the in-plane ferroelectric domains and the out-of-plane ferroelectric domains corresponding to the BaTiO_3_. A magnification of the in-plane and out-of-plane setup configurations is displayed in Figure S1 (Supporting Information). (c) X-ray reflectivity (XRR) of the Fe_3_O_4_/BaTiO_3_ heterostructure confirming a ∼20 nm thickness of the Fe_3_O_4_ film. (d) Representative reciprocal space map (RSM) along (*H* 0 *L*) planes (BaTiO_3_ reciprocal lattice units—r.l.u.), illustrating an incommensurate growth of the Fe_3_O_4_ thin film on the BaTiO_3_ substrate and (e) high-resolution projection on the BaTiO_3_ in-plane *H*-axis for an out-of-plane value of *L* = 2 (orange rectangle). (f) Optical image revealing distinct domain configurations, primarily composed of 90° domain walls forming striped regions oriented at 45° to the edges of the sample. Scale bar, 1 mm. (g) High-resolution optical image of the marked region in panel (f). Scale bar, 30 μm. (h) Domain mapping at the surface, and (i) the cross-section, corresponding to the area marked in panel (g). Scale bar, 30 and 10 μm. Raman images result from single depth-scan Raman spectra, with red indicating *a*-domains (in-plane) and blue indicating *c*-domains (out-of-plane), and intensity reflecting Raman intensity. The cyan color in panel (i) represents the localization of the Fe_3_O_4_ thin film. (j) atomic force microscopy (AFM) image of the surface matching panel (i). Scale bar, 10 μm. A line profile extracted from the AFM image reveals topographical features associated with domain boundaries. (k) Line profile from the AFM image showing topographical features and a schematic of the BaTiO_3_ unit cell illustrating domain orientations. The topography reveals the domain boundary topography associated with an asymmetric saw–teeth transition. The numerical annotations adjacent to both the Raman image in the cross-section and the AFM image delineate identical regions, with the cyan arrow indicating the topographical features of the Fe_3_O_4_ thin film. (l) AFM image highlighting the topography of the Fe_3_O_4_ thin film [cyan arrow in panel (k)]. (m) Line profile extracted from the AFM image showing the surface roughness to be less than 2 nm. (n) Schematic representation of the multiferroic Fe_3_O_4_/BaTiO_3_ heterostructure.

A BaTiO_3_ single crystal with a preferential (100) orientation (*a*-plane) measuring 5 × 5 × 0.5 mm was grown through top-seeded solution growth (TSSG) and provided by SurfaceNet GmbH (Germany). The crystal was polished using 1 μm diamond paste and subsequently cleaned with acetone and ethanol before characterization. To prevent nanoroughness-induced topographic artifacts, no further thermal or chemical etching was employed to reveal the domain structure. The Fe_3_O_4_/BaTiO_3_ heterostructure was fabricated by directly depositing Fe_3_O_4_ on untreated BaTiO_3_ (100) substrates. The substrates were characterized to confirm the presence of an approximately 2 nm native oxide layer. The deposition was performed using pulsed laser deposition (PLD) with a polycrystalline Fe_3_O_4_ stoichiometric target. A Nd:YAG laser with a 355 nm wavelength and a 2 Hz frequency was employed. To maintain the Fe_3_O_4_ stoichiometry, the deposition was carried out under an oxygen partial pressure of 3 × 10^–6^ mbar on a base ultrahigh vacuum pressure of 4 × 10^–8^ mbar. The substrate temperature was held at 773 K to encourage the crystalline growth of Fe_3_O_4_. Due to this step, the resulting BaTiO_3_ single crystal has slightly changed the ferroelectric domain distribution to alternated (100) and (001) orientations, as can be observed in Figure 1h–n.

### 2.2 Functional Properties Characterization

#### 2.2.1 Domain Structure Characterization

Domain mappings were conducted using a confocal Raman microscope (Witec alpha-300R) with a 532 nm excitation laser (intrinsic Raman light source) and a 100× objective lens. The confocal microscope offered lateral and vertical resolutions of approximately 250 and 500 nm, respectively, with a Raman mode spectral resolution of 0.02 cm^–1^. The domain walls of the samples were optically aligned perpendicular to the *x*-axis and parallel to the *y*-axis of the piezo-driven scan table. To generate the movement of the ferroelectric domain walls it was necessary to apply another laser diode light source coupled to the setup. This additional laser light was focused on the sample surface, parallel to the surface. Specifically, a 532 nm wavelength laser diode (Thorlabs, Inc.) was utilized at a power of 50 mW and a light spot diameter of ∼2 mm (perpendicular to the *y*-axis). The reversibility of light switching on ferroelectric domains during off–on–off light cycles was investigated. To do that, the process started with the confocal Raman microscopy (CRM) scanning of the sample surface under dark conditions (pristine state). Upon completion of one-third of the total scanning, the iris of the optical setup was opened, and the next third of the scanning was collected. Subsequently, the iris was closed, and the remaining third of the scanning was obtained. Raman data were processed by the Witec Control Plus Software.

#### 2.2.2 Topographic Information

Topographic images were obtained using the integrated AFM tip of the Witec alpha-300RA Raman microscope. AFM probes made of gold-coated silicon (NSG30 model, NT-MDT, Russia) were employed. These probes had dimensions of about 14–16 μm in height, aspect ratios ranging from 3:1 to 5:1, and curvature radii of 10 nm. Cantilevers used were 125 μm long, 40 μm wide, and 4 mm thick, with an elastic constant of 40 N/m and a resonance frequency of 268 kHz. Topographic images were acquired by scanning 256 lines with 512 points each over an area of 150 × 75 μm. The AFM operated in noncontact mode with a feedback set point at 50% of the free oscillation amplitude at the resonant frequency.

#### 2.2.3 Magnetic Characterization

Room temperature magnetic measurements were acquired with an alternating gradient force magnetometer (AGFM, PMC MicroMag 2900 Series AGM) at room temperature with the magnetic field applied parallel to the surface of the sample.^37^ The probes used to measure the sample guarantee a sensibility below 1 μemu, limited by background noise. The resolution in the field applied to the sample is below 1 Oe. Magnetic measurements were conducted in the plane of the films while rotating the sample to investigate the in-plane magnetic anisotropy. A 532 nm wavelength laser light was orthogonally directed onto the samples with a light spot ∼2 mm in diameter. The reversibility of light switching on magnetic hysteresis loops during off–on–off light cycles was studied, maintaining *M*_s_ constant in dark conditions, while at light power levels ranging from 5 to 50 mW the *M*_s_ signature is modulated. Low-temperature magnetic behavior to observe the magnetostructural coupling was examined with an MPMS-XL magnetometer. *M*_s_(*T*) data were collected in the thermal range comprised between 5 and 400 K at 3 kOe.

#### 2.2.4 Thermal Characterization

The thermal behavior of the Fe_3_O_4_/BaTiO_3_ heterostructure was assessed using a thermal camera (FLIR Systems T440) to monitor the sample temperature under illumination. Data acquisition and processing were performed using FLIR Tools+ software. In situ temperature measurements were initiated from the light-off state, with the optical system’s iris opening to expose the sample to light. The camera continued recording temperature changes until the completion of AFM measurements. Measurements were exported to data files using FLIR Tools+ and further processed with MATLAB software.

#### 2.2.5 Macroscopic Strain Measurement

Light-induced deformation was quantified using a WayCon inductive position transducer conditioned with a Solartron OD5 Module. The system had a linear scale of 42 V/mm, enabling the measurement of tens of nanometers with a high-resolution oscilloscope (Keysight DSOX2004A). For measurements under illumination, a 532 nm wavelength laser diode (Thorlabs, Inc.) was incorporated into the system. Light power was controlled using a source-measured unit (Keithley 2400) and set to 50 mW. The light spot had a diameter of ∼2 mm, and the duration of light irradiation was regulated using a switch. Raw data were processed to eliminate slow thermal drift, and a Savitzky–Golay filter was applied to smooth the data.

#### 2.2.6 Synchrotron Radiation High-Resolution XRD

XRD measurements were conducted at the SpLine CRG BM25 beamline at the ESRF The European Synchrotron (Grenoble, France) under the proposal MA-5601. The X-ray beam had a wavelength of 0.8266 Å (approximately 15 keV) with an energy resolution of Δ*E*/*E* ∼ 1 × 10^–4^. A beam spot size of 0.5 mm × 0.5 mm was used to ensure representative measurements of the crystalline domain structure. A lanthanum hexaboride (LaB_6_) standard was employed for instrument calibration and wavelength refinement. Measurements were carried out in reflection geometry using a SixC diffractometer in a vertical configuration. XRD patterns were recorded over an angular range of 5–25° (2Θ) with a step size of 0.0075° using a 2D photon-counting X-ray 2D-MAXIPIX detector.^38^ Reciprocal space maps (RSMs) were measured with the consideration of BaTiO_3_ lattice parameter units. All XRD data were processed using the BINoculars software.^39^ For measurements under illumination, a 532 nm wavelength laser diode (Thorlabs, Inc.) was coupled to the measurement stage. Light power was set to 30 mW, and the light spot diameter was approximately 2 mm. Light irradiation time was controlled using an electronic switch.

#### 2.2.7 X-ray Photoelectron Spectroscopy

X-ray photoelectron spectroscopy (XPS) data were acquired by utilizing a SPECS GmbH system, which featured a hemispherical energy analyzer PHOIBOS 150 9MCD. An Al X-ray source, operating at a power of 200 W and a voltage of 12 kV, was employed. Fe_3_O_4_/BaTiO_3_ heterostructure was initially placed within the prechamber at room temperature and subjected to degassing for several hours before being transferred to the analysis chamber. Pass energies of 50 and 20 eV were employed for collecting both survey and high-resolution spectra, respectively. In the case of measurements conducted under illumination, a 532 nm wavelength laser diode (Thorlabs, Inc.) was integrated into the measurement setup. The light power was set to 20 and 50 mW, with an approximate light spot diameter of 2 mm. Light irradiation duration was precisely controlled using an electronic switch. To evaluate the light’s effect on Fe_3_O_4_, we utilized a high-purity Fe_3_O_4_ powder (Sigma-Aldrich) as a reference and repeated the experiment both in the absence and presence of light.

## 3 Results and Discussion

### 3.1 Multiferroic Fe_3_O_4_/BaTiO_3_ Heterostructure

A Fe_3_O_4_-thin film is epitaxially grown onto a BaTiO_3_ crystal featuring a precisely controlled domain structure. This growth results in the establishment of an oriented array encompassing in-plane and out-of-plane domains within the Fe_3_O_4_ and BaTiO_3_ components, as confirmed by high-resolution synchrotron XRD analysis (Figure 1a,b). In the studied region, there is a preferential (100) orientation of the substrate, where the BaTiO_3_*a*-axis lies perpendicular to the substrate surface, while the *b*- and *c*-axes are contained in the substrate surface plane. The in-plane Θ–2Θ scan reveals the presence of highly crystalline twin boundaries along the *b*, *c*-axis of the BaTiO_3_ crystal. In contrast, the out-of-plane Θ–2Θ scan shows a single predominant domain along the *a*-axis. Regarding the Fe_3_O_4_ layer, structural stress effects are evidenced, since the interplanar distances obtained by in-plane and out-of-plane measurements are slightly different. Therefore, a strained cubic inverse spinel structure is observed, probably coming from crystal coherence and mismatch close to 4.7% with the substrate.^40^ The inferred cell parameters are *a*_∥,Fe_3_O_4__ = 8.48752(1) Å and *a*_⊥,Fe_3_O_4__ = 8.33312(1) Å. Despite the deformation, Fe_3_O_4_ retains approximately the volume of the unit cell (∼600 Å^3^ according to the calculated lattice parameters, being 592 Å^3^ for bulk samples).

XRR and RSM data provide additional corroboration of the highly precise growth obtained across the entire thickness of the layer (∼20 nm) on the crystal (∼500 μm), obtaining the Fe_3_O_4_/BaTiO_3_ heterostructure (Figure 1c–e). Specifically, a representative RSM along the *HL*_plane (*K* = 0) is displayed in Figure 1d where an incommensurate growth is observed. Structural relationships fulfill the out-of-plane *a*(Fe_3_O_4_)//*a*(BaTiO_3_) and in-plane *b*,*c*(Fe_3_O_4_) axis-on-axis with *b*,*c*(BaTiO_3_). Twin boundaries are also identified in every single BaTiO_3_ reflection (integer numbers) along the *c*-axis (*H*-in-plane). The noninteger reflections correspond to the strained Fe_3_O_4_ layer, and a projection along the in-plane direction is also shown for clarity. Figure 1f showcases an optical image of the Fe_3_O_4_/BaTiO_3_ heterostructure, wherein the transparency of the Fe_3_O_4_ thin film unveils the underlying ferroelectric domain pattern. The optical visualization unequivocally highlights the presence of an ordered arrangement of in-plane (marked in red) and out-of-plane (signaled in blue) ferroelectric domains (Figure 1g–n), findings that are consistently affirmed by the comprehensive analysis conducted through CRM and AFM (Figure 1h–m). Figure 1j,k shows the formation of an adequate domain structure containing charged domain walls which seems to be mandatory for achieving a reversible visible-light-controlled strain in ferroelectrics.^7^ Building upon the topographical imprinting of the BaTiO_3_ substrate onto the Fe_3_O_4_ layer (i.e., mirroring the stripe-like morphology of the array of *a*- and *c*-type ferroelectric domains), we embark on an exploration of changes in the magnetic properties of the system.

### 3.2 Impact of Light on the Magnetic Properties of the Fe_3_O_4_/BaTiO_3_ Heterostructure

**Figure 2:**
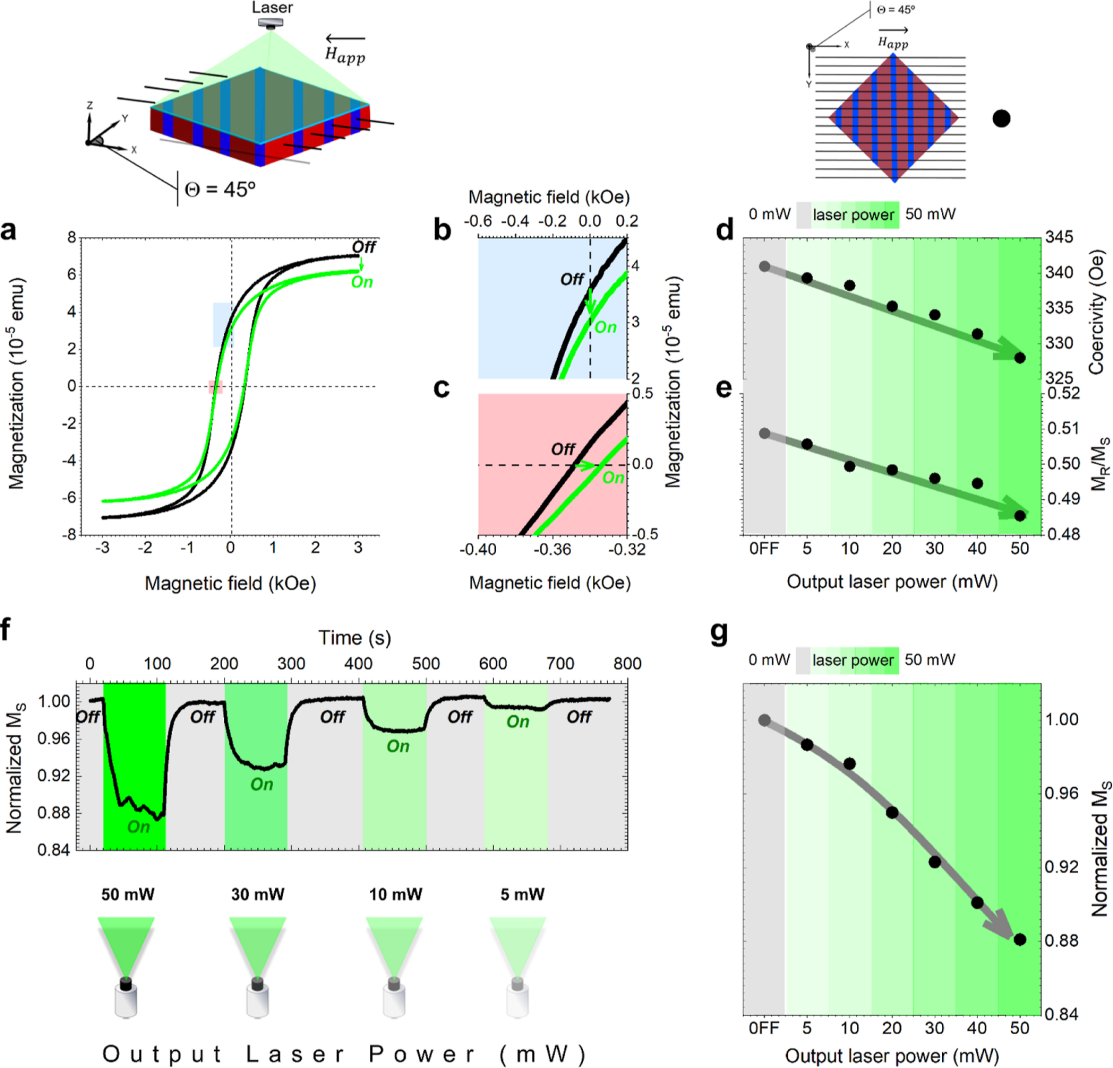
Magnetic response of the multiferroic Fe_3_O_4_/BaTiO_3_ heterostructure before and after Illumination. (a) Magnetic hysteresis loops are acquired both before and after illumination (50 mW of light power) along Θ = 45°. The ferroelectric domain distribution is presented at the top Figure along with the corresponding angular nomenclature used for characterizing the magnetic properties. Panels (b,c) provide a magnified view of the hysteresis loops taken along the diagonal direction (45°), highlighting alterations in the coercivity (*H*_C_) and remanence ratio (*M*_r_/*M*_s_) of the Fe_3_O_4_/BaTiO_3_ heterostructure. (d,e) Evolution of the *H*_C_ and *M*_r_/*M*_s_ as a function of the light power. At the top of panels (d,e) a 2D schematic representation of the measurement conditions representing the values obtained for the 45° configuration. (f) A sequence of *M*_s_ taken along the diagonal direction (45°) during an off–on–off light succession, and (g) evolution of normalized *M*_s_ with varying light power. The dashed black line of the panel (g) delineates the value of *M*_s_ in the absence of illumination, which, in our case, equals 1, as the values have been normalized to these dark conditions. The reversible nature of this phenomenon is demonstrated, as the optically induced change in the *M*_s_ disappears after the light is switched off. A simplified scheme representing the measurement sequence is presented below, where the light is switched on/off at the positions indicated by green/gray regions.

We systematically scrutinize the magnetic behavior of the Fe_3_O_4_/BaTiO_3_(100) heterostructure under two distinct conditions: in the absence and under a green laser irradiance of 50 mW. Figure 2a shows the magnetization curves of the system corresponding to the laser switched on and off in a Θ = 45° configuration (i.e., ferroelectric domain walls are perpendicular to the applied magnetic field). The off-curve shows a robust hysteretic signature and a relatively soft coercivity (*H*_C_) of about 340 Oe. This value is slightly higher than those found in other works, probably due to the 4.7% mismatch needed to balance approximately 2 times the BaTiO_3_ lattice parameters.^41^ Upon 50 mW laser illumination, saturation magnetization (*M*_s_) remarkably drops by ∼12%. The effect also manifests itself in the *H*_C_ and remanence ratio (*M*_r_/*M*_s_), which decreases by ∼5% (Figure 2b,c). To understand this substantial light-induced alteration in the magnetic behavior of the Fe_3_O_4_/BaTiO_3_ heterostructure, we address a series of experiments to examine the magnetic dependency on light power, as depicted in (Figure 2d–f). *H*_C_ and *M*_r_/*M*_s_ decrease proportionally as a function of the output laser power (Figure 2d,e) and do so in a discrete manner with values between 4 and 5% respectively, but with a distinct linear trend. However, *M*_s_ drops up to ∼12% following a decreasing exponential trend, which may suggest that other mechanisms or couplings between their physical properties (magnetic, electrical, and/or structural) are involved, different from those causing the changes in *H*_C_ and *M*_r_/*M*_s_. In this scenario, sequential off–on light cycles (Figure 2f) showcase the reversibility of the light-induced *M*_s_ changes with the profiles corresponding to the off–on states. In addition, the magnitude of the change depends on the power of the incident light (Figure 2g).

As portrayed in Figure 2, we also measure in-plane hysteresis loops applying the magnetic field along two additional sample rotation angles Θ = 0° and, and Θ = 90° (Figure S2 in the Supporting Information), both dark and under illumination. Interestingly, a progressive decrease in *H*_C_ and *M*_r_ is also noticed when we switch to the configuration of Θ = 0° and 90° respectively, indicating clear easy and hard axes of magnetocrystalline anisotropy within a uniaxial framework.^19^ Given that Fe_3_O_4_ usually presents biaxial magnetocrystalline anisotropy, the uniaxial character displayed here strongly supports the existence of magnetoelectric coupling between the ferroelectric and ferrimagnetic domains, as in fact uniaxial anisotropy has been reported in CoFeB magnetic layers magnetoelectrically coupled to BaTiO_3_.^42^ Specifically, it is worth noting as well that the observed effects are most pronounced in *M*_s_ when the applied magnetic field is oriented at 45° (on-curves in Figure S2 in the Supporting Information). Irreversible variations in magnetic properties have been recently demonstrated in heterostructures composed of a 40 nm Ni metal film on BaTiO_3_ via visible light illumination at 100 K.^43^ The novelty here is underscored by the consistent and reversible control and modulation of a macroscopic magnetic response that is both discernible and readily monitorable at room temperature.

### 3.3 Unveiling Light-Induced Mechanisms Modulating Magnetic Properties

The variations in magnetic behavior induced by light can be explained by three distinct scenarios or a combination thereof. First, it builds upon our previous studies in which we demonstrated the possibility of exerting control over strain through light-induced mechanical deformation in the BaTiO_3_ substrate. Given that the macroscopic strain induced by light in BaTiO_3_ primarily originates from the light-driven motion of domain walls, an anisotropic strain is anticipated, provoking magnetostriction at the ferrimagnetic/ferroelectric Fe_3_O_4_/BaTiO_3_ interfaces and resulting in the variation in *H*_C_ and *M*_r_. However, this perspective alone (that is, magnetostriction) does not account for the observed variation in *M*_s_ because in this magnetic state the spins are fully aligned with the applied magnetic field (*H*_app_). Second, the Fe_3_O_4_/BaTiO_3_ heterostructure displays a strong magnetoelectric coupling^20,40,41,44^ originated from interface bonding sensitive to atomic displacements at the interface, with the appearance of ferroelectric domain walls electrostatic decompensations, which in turn lead to changes in the interface magnetization upon reversal of the ferroelectric layer’s polarization. This second scenario may require an electric circuitry to apply electric fields capable of causing a change in bonding properties between Fe and Ti atoms, inducing magnetic moments on the interface Ti atoms sensitive to the Fe–Ti bond length.^44^ Therefore, this mechanism could provide a plausible explanation for the behavior observed in *M*_s_. However, there are precedents in metallic magnetic systems (Fe/BaTiO_3_) in which a magnetic dead layer is formed with the applied electric field that results in irreversible magnetic changes, decreasing its original magnetoelectric coupling.^23^ Lastly, the third scenario, and the simplest scenario, considers Joule heating effects generated by the laser application as a potential explanatory mechanism for the observed magnetic behavior variations. These perspectives offer a comprehensive framework to understand the observed effects, requiring further scrutiny to discern the relative contributions of each mechanism to the observed phenomenon.

**Figure 3:**
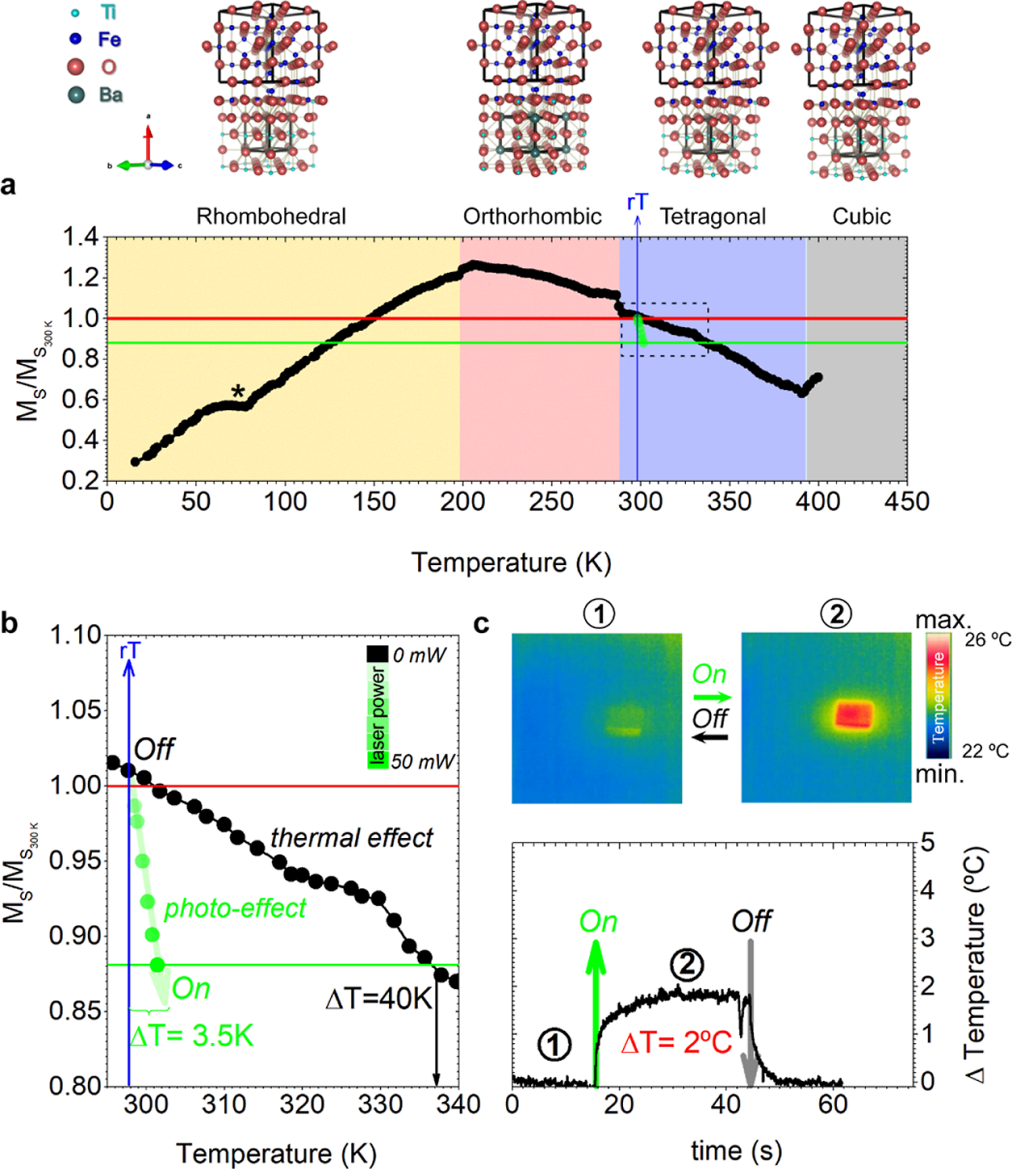
Ruling out warming as a physical mechanism responsible for the light-reversible magnetic responses on the Fe_3_O_4_/BaTiO_3_ heterostructure. (a) In-plane *M*_s_/*M*_s,300K_(*T*) behavior of the Fe_3_O_4_/BaTiO_3_ heterostructure in the absence (black solid circle) and presence of illumination (green solid circle) with an applied magnetic field of 3 kOe. In top, panel (a) shows the structural phase transition regime of the coupling at the ferrimagnetic/ferroelectric Fe_3_O_4_/BaTiO_3_ interfaces, respectively. The asterisk near 80 K represents a possible weak Verwey transition corresponding to the Fe_3_O_4_ layer. The blue arrow represents the values of *M*_s_/*M*_s,300K_ at room temperature (rT), while the red and green lines represent the values of *M*_s_/*M*_s,300K_ equal to 1 and the light-induced values, respectively. (b) A magnified view of comparative representation of the *M*_s_/*M*_s,300K_(*T*) behavior of the Fe_3_O_4_/BaTiO_3_ heterostructure (thermal effect, black solid circle) and the light-induced *M*_s_ (green solid circle), revealing that a variation of normalized *M*_s_ of ∼12% due to a thermal effect occurs for an Δ*T* = 40 K, while the same variation induced by light generates a heating of an order of magnitude less, that is Δ*T* = 3.5 K. The scale of the light power density was adjusted to easily compare the light power and thermal effect needed to produce the same normalized *M*_s_ on the sample. (c) Thermal camera images at different measurement times and labeled as 1 and 2 states. The incident light has a power of 30 mW and a wavelength of 532 nm. Time dependence of the temperature evolution of the sample. States 1 and 2 indicate a situation when the light switches off and on, respectively. The temperature is plotted relative to rT.

To unravel the underlying physical mechanism responsible for the observed effects and confirm the role of magnetoelectric coupling at the interface, first we will rule out the Joule heating effect as the driving force (Figure 3). To eliminate this potential scenario, we systematically investigate the magnetization versus temperature and *M*_s_(*T*) behavior of the Fe_3_O_4_/BaTiO_3_ heterostructure under dark conditions (Figure 3a). It is unequivocal that the magnetization evolution of the Fe_3_O_4_ is influenced by the system’s temperature since it follows the structural transitions of the BaTiO_3_^20^ (a magnification of the simulated crystal structures for BaTiO_3_ as a function of temperature is added for clearness in Figure S3 in Supporting Information).

Intriguingly, a magnetic anomaly is detected around 80 K (marked with an asterisk in Figure 3a), which could be associated with a weak Verwey transition.^45^ A fact that probably stems from its epitaxy with the substrate and proves its magnetostructural coupling with the layer. However, upon closer examination, it becomes clear that the Joule heating required to generate the observed light-induced effects is markedly distinct from the temperature conditions considered in the absence of illumination (Figure 3b). While variations in the system’s temperature undeniably influence its magnetization, the Joule heating generated by the laser illumination is measured to result in a temperature increase of 3.5 K (Figure 3b,c). To put it plainly, we would need an order of magnitude higher heating (∼40 K) to achieve a comparable variation in magnetization through the Joule effect alone (Figure 3b). This fact emphasizes the uniqueness of the light-induced magnetic effects we have observed and underscores the imperative need for a comprehensive understanding of the intricate interplay between light, strain, and polarization in this multiferroic heterostructure.

**Figure 4:**
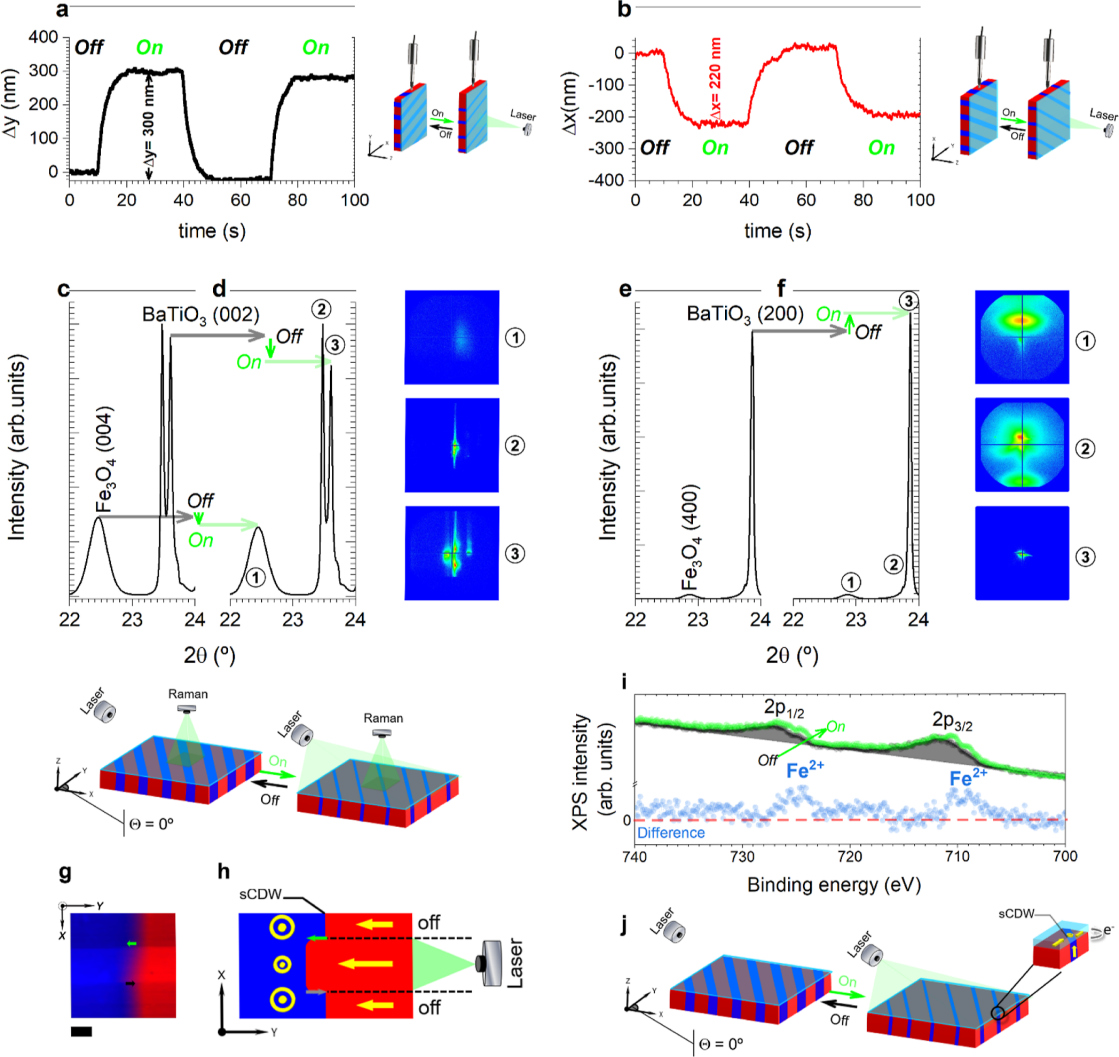
Mechanisms governing light-induced control of magnetic properties in the Fe_3_O_4_/BaTiO_3_ heterostructure. (a,b) Macroscopic deformation induced by light in the direction perpendicular (a) and parallel (b) to the BaTiO_3_ domain stripes. Noticeable elongation occurs perpendicular to the domain walls, while slight contraction is observed parallel to them. Two cycles of light on–off ensure measurement repeatability. Measurement and experimental schemes are depicted above the plots. (c,d) High-resolution in-plane and (e,f) out-of-plane XRD patterns before (c,e) and after illumination (d,f). XRD reflections correspond to the 002 and 200 of the BaTiO_3_ substrate and the 004 and 400 of the Fe_3_O_4_ thin film. These patterns unambiguously illustrate the impact of light on the relative intensity of in-plane ferroelectric domains (c,d) and out-of-plane ferroelectric domains (e,f) within BaTiO_3_. The green and black arrows denote the moments of light activation and deactivation, respectively. The images obtained with the 2D-MAXIPIX detector in different measurement windows during the XRD scan (1-Fe_3_O_4_ layer, 2-BaTiO_3_ substrate, and 3-BaTiO_3_ substrate) are added to the right of the figure as an example under illumination conditions for the in-plane configuration (c,d) and out-of-plane configuration (e,f). Twin boundaries are observed in 2 and 3 shots along the in-plane direction in (c,d). (g) CRM image and (h) representative scheme evidencing the reversibility of light switching on ferroelectric domains during off–on–off light cycles, operating at 50 mW of light power. Highlight that the in-plane ferroelectric domains are represented in red, while the out-of-plane domains are depicted in blue. Scale bar, 600 nm. The green and black arrows denote the moments of light activation and deactivation, respectively. (i) Fe 2p core level measurements before and after illumination. (j) Simplified scheme illustrating the light-induced motion of domain walls during the illumination process, highlighting the light-induced *c*- to *a*-domain switching and the consequent modification of their vector polarization. The vectors of polarization within the plane (in the red region) are depicted as yellow arrows, while those outside the plane (in the blue region) are denoted by yellow dots.

To comprehend the variations in magnetic behavior induced by light on the Fe_3_O_4_/BaTiO_3_ heterostructure (*M*_r_, HC, and *M*_s_), we need to scrutinize the changes in the ferroelectric domain structure and the macroscopic elongation and local strain of the BaTiO_3_ substrate. The investigation is prompted by our prior exploration of visible-light-induced deformation in BaTiO_3_, which demonstrated the feasibility of controlling the strain by visible light in a ferroelectric crystal.^7,8,46^ Here, we observe a remarkable macroscopic elongation of approximately 300 nm along the *Y*-direction (Figure 4a), accompanied by a contraction of about 200 nm in the *X*-direction (Figure 4b). In relative terms, the photostrain along the *Y*-axis is +0.006%, while the contraction along the *X*-axis is −0.004%. The macroscopic deformation of the BaTiO_3_ crystal originates from the collective motion of the ferroelectric domain walls, leading to a domain rearrangement when the material is exposed to light.^47^ This marked deformation anisotropy provides an additional finding that the thermal contribution is residual since if it were a purely thermal effect, the Fe_3_O_4_/BaTiO_3_ heterostructure would expand in all directions. Likewise, to provide further empirical support for the occurrence of ferroelectric domain switching, we conducted an analysis of the XRD pattern of BaTiO_3_ under illumination conditions (∼30 mW). As depicted in Figure 4c,d, the impact of light on the relative intensity of the (002) in-plane peaks associated with *c*-domains reveals a significant decrease of 16.4(5)%. Conversely, this light-induced effect results in a relative increase of the out-of-plane ferroelectric *a*-domains by 7.5(1)% (Figure 4e,f). This transition from ferroelectric *c*-domains to *a*-domains is substantial and attributed to the optically driven motion of domain walls.^7,8,46^ A comprehensive analysis in the Fe_3_O_4_ thin film mirrors these variations, with slight changes in in-plane and out-of-plane crystalline domains of −3.6(3)% and +1.7(1)% respectively, consistent with the BaTiO_3_ crystal (see calculated values in Table S1 in the Supporting Information). Importantly, these variations are reversible under light and do not result in a loss of crystalline coherence (epitaxy), as observed in the RSM conducted on the same HL section of Figure 1d under light conditions (Figure S4 in the Supporting Information). This result underscores the robust epitaxial relationship between these two materials with such structural displacements potentially influencing the magnetic response of the Fe_3_O_4_/BaTiO_3_ heterostructure. Furthermore, examination via CRM mapping demonstrates that light stimulation leads to a relative increase in a-domains in specific regions (Figure 4g,h), attributed to the motion of domain walls driven by optical excitation. The reversibility of this domain rearrangement is illustrated through sequential off–on–off light cycles.

The two main magnetoelectric coupling mechanisms are strain-mediated and charge-mediated effects.^41,44,48^ On the one hand, strain-mediated effects based on applying external electric fields to a piezoelectric substrate and the magnetostriction of a magnetic overlayer have been reported to modulate the magnetization of Fe_3_O_4_/BaTiO_3_ heterostructures at low magnetic fields, typically by amounts of the order of 1% under electric fields of 400 kV/m.^35,36,41^ We, therefore, expect the light-induced macroscopic elongation and local strain of BaTiO_3_ to influence the observed variations in *H*_C_ and *M*_r_ through magnetoelastic coupling.^21^ However, the magnetoelastic coupling cannot account for the variations in *M*_s_, which are actually 1 order of magnitude larger (∼12%) than the changes in *H*_C_ and *M*_r_/*M*_s_. Indeed, inverse magnetostriction may lead to rotation of the easy magnetic axis and changes in interatomic distances and resulting exchange interactions, but neither of these two effects is reflected in *M*_s_.^44^ In fact, the primary sources of magnetic anisotropy changes, including magnetoelasticity, magnetostatics, magnetocrystalline properties, and exchange, are deemed negligible under saturated conditions. This consideration is based on the minimal reversible structural variations observed in the Fe_3_O_4_ layer under light conditions (Figure 4d–f). Consequently, it becomes evident that another mechanism, stemming from a strong magneto-electric coupling, indeed underlies the modification of *M*_s_.

On the other hand, charge-mediated mechanisms have also been demonstrated to generate robust magnetoelectric coupling, also in Fe_3_O_4_/BaTiO_3_ heterostructures and typically triggered by applying external electric fields to the sample. As a consequence of these electric fields, ferroelectric domain movements lead to local electric polarization and atomic displacements that are induced at the interface.^16,44,49,50^ The magnetoelectric effect, specifically the converse, involves a variation of magnetization (*M*) due to the application of an electric field (*E*) according to the relation Δ*M* = α_C_·Δ*E*,^51^ where α_C_ represents the magnetoelectric coupling coefficient. Studies have demonstrated significant modifications in the magnetic properties of multiferroic structures with the application of an electric field, including *M*_s_ modulations of up to 10%.^16,23,49^ In our case, the incidence of light modifies the domain wall compensation such that the depolarization field inside each domain changes, thereby triggering the domain wall motion.^46^ Likewise, the charge-mediated mechanism is claimed to generate an electric polarization at the interface that leads to changes in the oxidation and spin state of the Fe cations, which are responsible for the variations in *M*_s_.^34,35^ To verify this, we conducted XPS measurements on the Fe_2p_ core level to identify potential electronic variations in the Fe^2+^ and Fe^3+^ populations on the surface. Such variations may indicate a redistribution of the BaTiO_3_-induced charge (Figure 4i). Fe_3_O_4_ is a mixed-valence iron oxide, where the tetrahedral positions of Fe are occupied by +3 cations and the octahedral positions alternate between +2 and +3 cations.^25^ Surprisingly, the application of 50 mW green light results in a significant variation in the Fe^2+^ and Fe^3+^ populations at the surface, with a clear predominance of the Fe^2+^ contribution. This leads to an accumulation of negative charge on the surface, causing variation in the net magnetic moment within the ferrimagnetic structure of Fe_3_O_4_. Specifically, the magnetic moment of Fe^3+^ in the tetrahedral positions is antiparallel to Fe^3+^ and Fe^2+^ in the octahedral positions in the same ratio. In contrast, the spins in the octahedral positions are distributed in a parallel arrangement. Therefore, the net magnetic moment results from the magnetic moment of Fe^2+^ (4 μB).^52^ The observation that *M*_s_ is devalued with light, accompanied by an excess of negative charge (Fe^2+^), suggests that the octahedral positions originally occupied by Fe^3+^ are partially depleted to Fe^2+^. The decompensation disrupts the antiferromagnetic lattice coupling with the tetrahedral positions of Fe^3+^. Our results strongly suggest that these light-induced changes in the Fe cation local environments are reversible, in agreement with previous studies using electric fields instead of light.^53^

To corroborate this hypothesis, we performed the same experiment on a commercial Fe_3_O_4_ powder pellet, as this material is a semimetal with a 0.14 eV band gap.^54^ This band gap could generate electron–hole pairs that modify the magnetic properties. The XPS difference pattern with and without light reveals negligible differences, conclusively demonstrating the charge-mediated magnetoelectric coupling origin of the reversible modulation of *M*_s_ (Figure S5 in Supporting Information).

## 4 Conclusions

Our study achieves advances in understanding and exploiting the functional capabilities of multiferroic oxide heterostructures, which respond reversibly to low-power laser light. This novel approach modulates the magnetic properties (*H*_C_, *M*_r_, and *M*_s_) of the Fe_3_O_4_/BaTiO_3_ heterostructure with no need to add circuitry or complexities related to electrical interfaces. Considering the perspective regarding the origin of changes in magnetic properties, this achievement is based on two mechanisms. First, the magnetoelastic effect, initiated by the ferroelectric domain switching (macroscopic structural deformation), causes a proportional change in the coercivity and remanence under laser excitation. Second, the interaction between light and matter triggers the electrostatic decompensation of charged ferroelectric domain walls, thereby influencing the magnetization of the epitaxial Fe_3_O_4_ layer through magnetoelectric coupling. The origin of these concurrent phenomena is attributed to the robust coupling between magneto-electric and structural elements within the heterostructure. This interaction, driven by light, strain, and polarization, shows advances in wireless control of magnetic properties in the field of multiferroic heterostructures.

## Supporting Information Available

S1, high-resolution XRD configurations for light-induced domain switching determination. S2, investigating light power dependency for robust light-induced magnetic control in multiferroic systems. S3, BaTiO_3_ crystal structures found with temperature. S4, investigating light power dependency for robust light-induced magnetic control in multiferroic systems. S5, impact of light on isolated Fe_3_O_4_ structure by XPS (PDF)
