# Supplementary material for: Concomitant Light-Reversible Magnetic Response in Multiferroic Oxide Heterostructures for Multiphysics Applications
Source: ACS Appl Mater Interfaces. 2024 Apr 8;16(15):19866–76. doi: 10.1021/acsami.4c02551 (PMC11040584; doi:10.1021/acsami.4c02551)
Supplement: Supplementary file 1 — am4c02551_si_001.pdf [file am4c02551_si_001.pdf]

SUPPORTING INFORMATION

Concomitant Light-Reversible Magnetic Response in Multiferroic Oxide Heterostructures for Multiphysics Applications

Jesús López-Sánchez,[1,*] Adolfo del Campo,[1] Adrián Quesada,[1] Alejandro Rivelles,[2] Manuel Abuín,[2] Raquel Sainz,[3] Eugenia Sebastiani-Tofano,[4,5] Juan Rubio-Zuazo,[4-5] Diego A. Ochoa,[6] José F. Fernández,[1] José E. García,[6,*] and Fernando Rubio-Marcos[1,*]

[1] Departament of Electroceramics, Instituto de Cerámica y Vidrio – Consejo Superior de Investigaciones Científicas (ICV – CSIC), 28049 Madrid, Spain.

[2] Instituto de Sistemas Optoelectrónicos y Microtecnología (ISOM), Universidad Politécnica de Madrid (UPM), 28040 Madrid, Spain

[3] Instituto de Catálisis y Petroleoquímica – Consejo Superior de Investigaciones Científicas, (ICP – CSIC), 28049 Madrid, Spain

[4] Instituto de Ciencia de Materiales de Madrid – Consejo Superior de Investigaciones Científicas (ICMM – CSIC), 28049 Madrid, Spain

[5] Spanish CRG BM25 – SpLine at the ESRF – The European Synchrotron, 38000 Grenoble, France

[6] Department of Physics, Universitat Politècnica de Catalunya (UPC), 08034 Barcelona, Spain

*Authors to whom correspondence should be addressed. J.L-S, J.E.G and F.R-M: Email: jesus.lopez@csic.es; jose.eduardo.garcia@upc.edu; frmarcos@icv.csic.es

S1 High-Resolution X-ray Diffraction Configurations for Light-Induced Domain Switching Determination.

Different diffraction conditions are required to study the ferroelectric domain switching of the $BaTiO_3$ crystal, always following Bragg's law. For this purpose, a six-circle diffractometer is used, whose geometry and motor motions are capable of monitoring the light reversibility of pure in-plane and out-of-plane ferroelectric domains. The instrumental design and thanks to the beam quality with a huge signal-to-noise ratio provided by The European Synchrotron (ESRF), valuable data is provided on the in-situ behavior of the $BaTiO_3$ single crystal and on the magnetostructural coupling with the Fe_3O_4 layer operated by light.

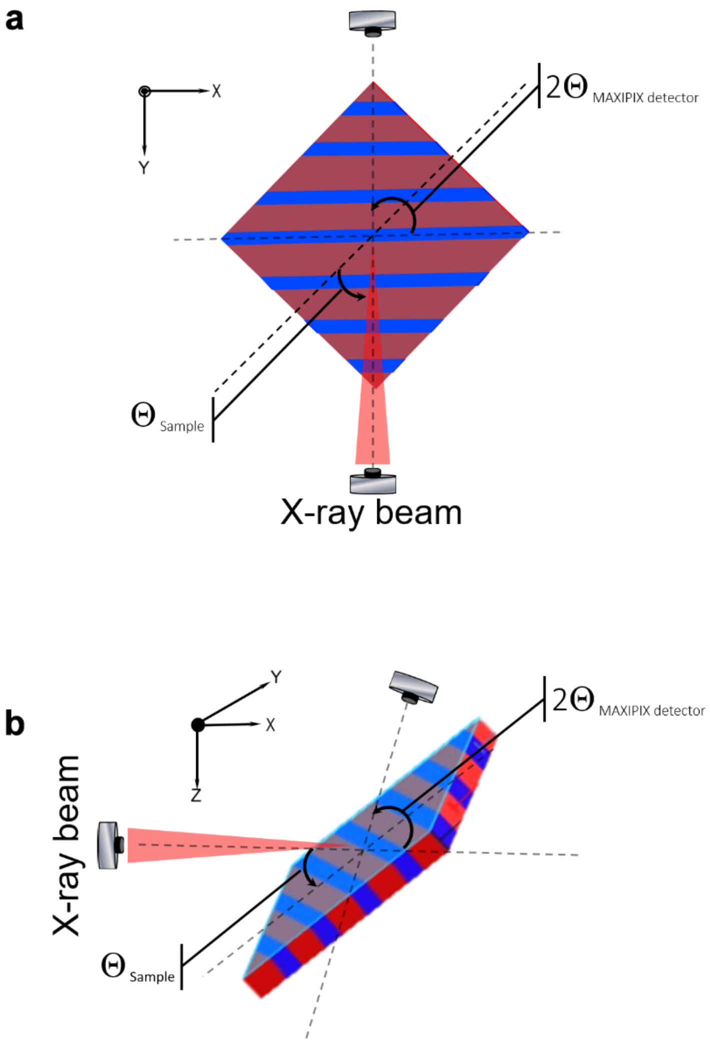

Figure S1: High resolution X-ray diffraction set-up configurations. a, In-plane and **b,** out-of-plane Θ-2Θ scans configurations performed by a six-circle diffractometer in the CRG SpLine Beamline BM25 in Grenoble, France.

S2 Investigating Light Power Dependency for Robust Light-Induced Magnetic Control in Multiferroic Systems.

As illustrated in **Figure S2**, we have conducted in-plane hysteresis loops while varying the laser power over a range from 5 mW to 50 mW. These measurements were carried out with the magnetic field aligned at different sample rotation angles, specifically at Θ=0° (**Figure S2a**), Θ=45° (**Figure S2b**), and Θ=90° (**Figure S2c**), under both dark and illuminated conditions. Significant findings from these foundational experiments confirm our ability to systematically regulate the magnetic behavior induced by light through controlled variations in light power. The observed relationship demonstrates a linear correlation between the magnetic behavior of the $Fe_3O_4/BaTiO_3$ heterostructure -that is, H_C, squareness (M_r/M_s), and saturation magnetization (M_s)-, and the applied light power across the examined range (see **Figure 2f-i** of the main manuscript.

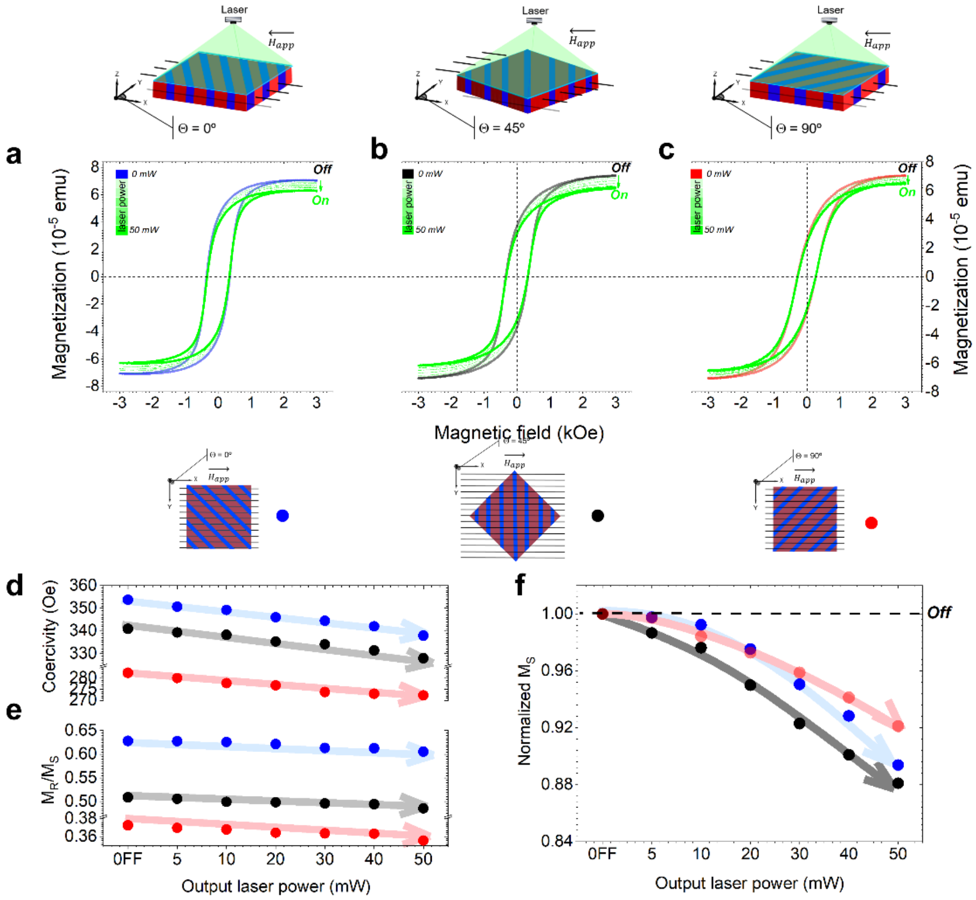

Figure S2: Magnetic Features of the Multiferroic $Fe_3O_4/BaTiO_3$ Heterostructure as a Function of the Output Laser Power. Magnetic hysteresis loops are collected by an alternating gradient magnetometer (AGM), applying light with a green laser diode (λ = 532 nm) from 0 to 50 mW along different sample orientations: **a**, Θ=0°, **b**, Θ=45°, and **c**, Θ=90°. A schematic of the ferroelectric domain distribution is displayed on top of each figure. A variation of the remanence and coercivity as a function of the sample rotation angle is observed for each output laser power, showing a uniaxial anisotropy caused by pulsed laser growth (PLD). **d**, **e**, and **f**, Evolution of the H_C, M_R/M_S), and normalized M_S, respectively, as a function of the light power. The dashed black line of the panel **h** delineates the value of Ms in the absence of illumination, which, in our case, equals 1, as the values have been normalized to these dark conditions. At the top of panels **f-g**, a 2D schematic representation of the measurement conditions is presented, with black, blue, and red dots representing the values obtained for the 0°, 45°, and 90° configurations, respectively.

S3 $BaTiO_3$ Crystal Structures Found with Temperature.

$BaTiO_3$ undergoes distinct structural transitions with varying temperatures. Below 200 K, it exhibits a rhombohedral structure, followed by an orthorhombic structure between 200 and 275 K. From 275 to 400 K, it displays a tetragonal structure, and above this value, it is cubic[1]. Notably, only the cubic structure displays paraelectric characteristics. The structural parameters used for the simulation of the crystalline structures that can occur as a function of temperature are detailed in **Figure S3**.

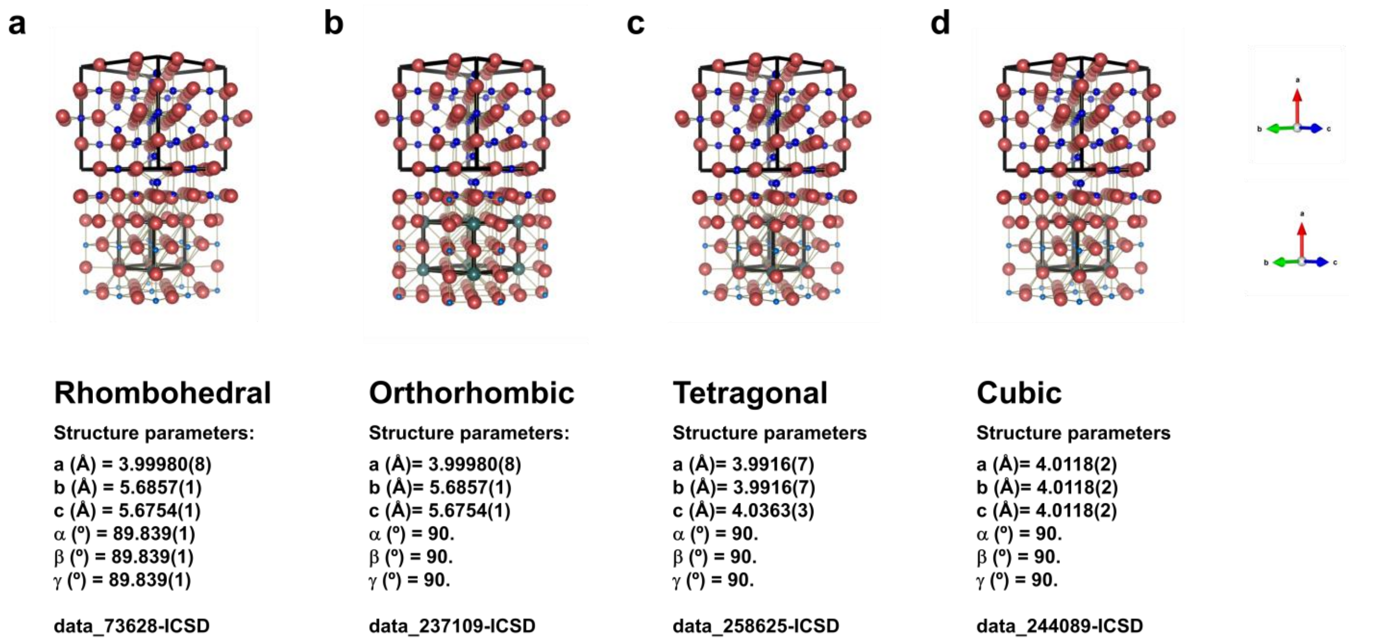

Figure S3: $BaTiO_3$ crystal structures depending on the thermal range. **a**, Rhombohedral, **b**, orthorhombic, **c**, tetragonal, **d**, cubic crystal structures of $BaTiO_3$ that are found in the thermal range <200 K, 200 K < T < 275 K, 275 K < T < 400 K and T > 390 K, respectively.

S4 Investigating Light Power Dependency for Robust Light-Induced Magnetic Control in Multiferroic Systems

It is essential to examine the variations in the crystalline domain and lattice parameters of the Fe_3O_4/$BaTiO_3$ heterostructure with light as they trigger relevant conclusions to properly predict the origin of the observed strong magnetic changes. Reciprocal space maps (RSMs) obtained in a representative H_L region are shown in **Figure S4** for K=0, applying an output laser power of 30 mW. As in **Figure 1** in the manuscript, an incommensurate growth of Fe_3O_4 on $BaTiO_3$ is evident where the integer positions correspond to the reflections of the $BaTiO_3$ crystal and the non-integer positions to the reflections of the Fe_3O_4 layer. The lattice parameter of Fe_3O_4 (8.396 Å)[2] is approximately twice the lattice parameter of $BaTiO_3$ (a=3.99916 Å and c=4.03630 Å).[1] Therefore, the observed reflections of Fe_3O_4 possess twice the order of the adjacent reflection corresponding to $BaTiO_3$. Note that twin boundaries are observed in all reflections found in this H_L region along the L direction. The figure on the right shows the H_projection of the orange delimited area where the $Fe_3O_{4,\ (8\ 0\ 4)}$ and $BaTiO_{3,\ (4\ 0\ 2)}$ reflections are displayed. Table S1 shows the retrieved structural parameters from the RSM data.

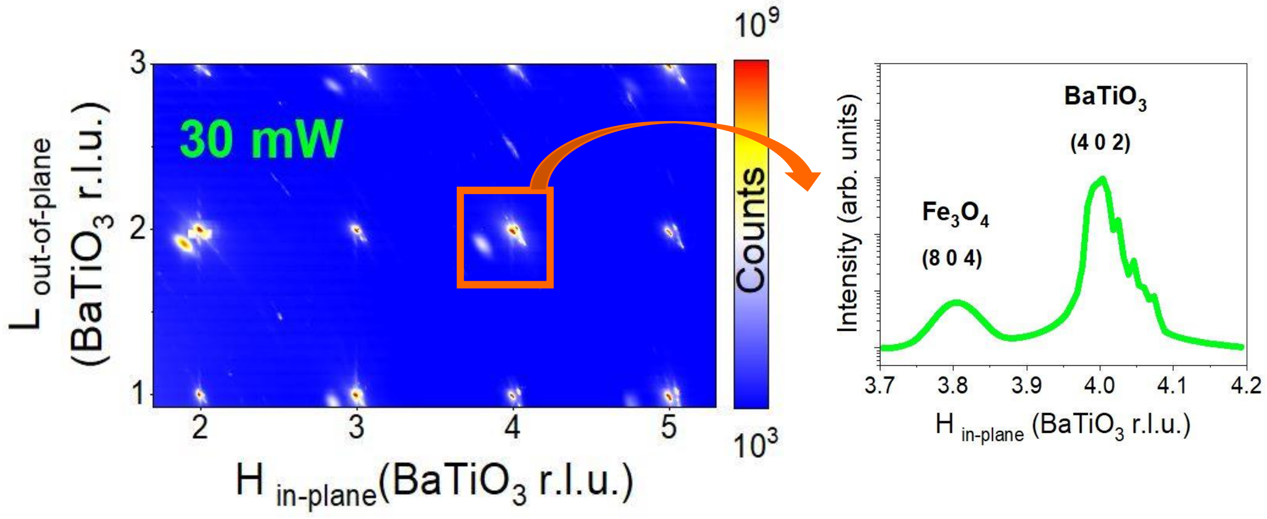

Figure S4: Stable Incommensurate Growth with Green Light Exposure. Reciprocal space map (RSM) acquired from representative reciprocal H_L region with K=0 (same region as **Figure 1d**, manuscript). Integer and non-integer numbers correspond to the $BaTiO_3$ and Fe_3O_4 reflections represented in $BaTiO_3$ reciprocal lattice units (r.l.u.), respectively. Indicated orange region is taken for H_in-plane, manifesting the epitaxy and good crystallinity of the Fe_3O_4 thin film.

Table S1: Calculated in-plane and out-of-plane crystalline domain and cell parameters of the Fe_3O_4 thin film retrieved from RSM analyses. Valuable differences can be observed in the crystalline domain size with light. However, variations in the lattice parameter are negligible, evidencing a dominant magneto-electric coupling.

Light Power	In-Plane Crystalline Domain, $d_{\parallel}$ (Å)	Out-of-Plane Crystalline Domain, $d_{\perp}$ (Å)	In-Plane Cell Parameter, $a_{\parallel}$ (Å)	Out-of-Plane Cell Parameter, $a_{\perp}$ (Å)
OFF	80.84(1)	167.87(1)	8.48752(1)	8.33312(1)
ON	77.95(1)	170.72(2)	8.48460(1)	8.33320(1)
Variation, Δ	-3.57(%)	+1.71(%)	-0.034(%)	0.001(%)

S5 Impact of Light on Isolated Fe_3O_4 Structure by XPS

To ascertain that the induced charge redistribution in $BaTiO_3$ plays a pivotal role in modifying electronic variations in the Fe^{2+} and Fe^{3+} populations on the surface of the Fe_3O_4 layer, we have devised an experiment. Herein, we assess the electronic structure through X-ray Photoelectron Spectroscopy (XPS) measurements on the Fe 2p core level, both in darkness and under the influence of light using a Fe_3O_4 powder sample. **Figure S5** illustrates that the XPS difference pattern, comparing measurements with and without light, reveals negligible differences. This unequivocally establishes the origin of the variation observed in M_S, which is a consequence of the magnetoelectric coupling between the $BaTiO_3$ and the Fe_3O_4 layer, generated by the induced charge redistribution in $BaTiO_3$.

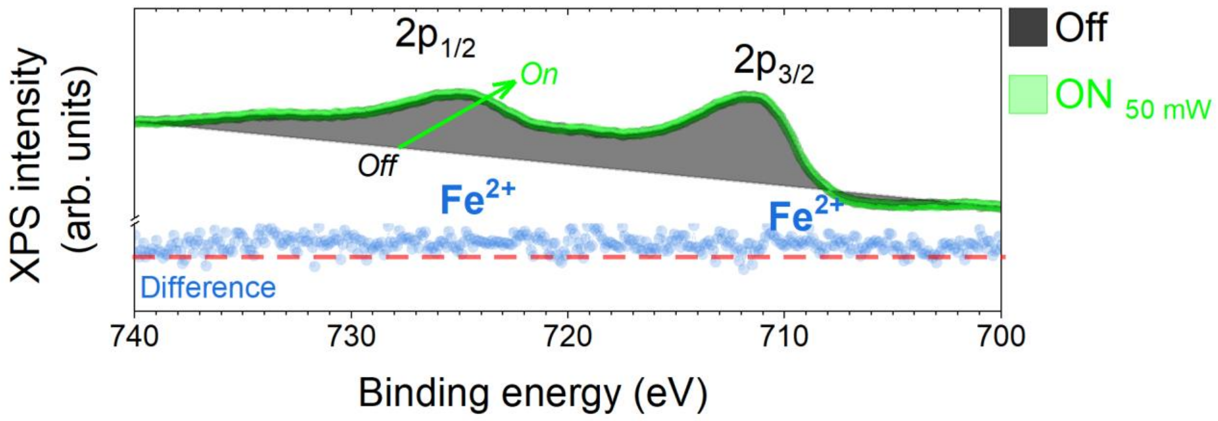

Figure S5. X-ray Photoelectron Spectra (XPS) – Fe 2p Core Level Measurements Before and After Illumination to Assess the Impact of Light on isolated Fe_3O_4 structure. The experiments were conducted using a high-purity Fe_3O_4 powder (Sigma-Aldrich) as a reference, with repetitions under both dark and illuminated conditions, with light to a power of 50 mW. Fe^{2+} indications (blue) show the regions approximately where its main contribution is located.

References

(1) Necib, J.; López Sánchez, J.; Rubio-Marcos, F.; Serrano, A.; Navarro, E.; Peña, Á.; Mnasri, T.; Smari, M.; Rojas Hernandez, R. E.; Carmona, N.; Marin, P. A Feasible Pathway to Stabilize the Monoclinic and Tetragonal Phase Coexistence in Barium Titanate-Based Ceramics. *J Mater Chem C Mater* **2022**, 17743–17756. https://doi.org/10.1039/d2tc04265g.

(2) Cornell, R. M.; Schwertmann, U. *The Iron Oxides*; Wiley: Weinheim; Germany, **2003**. https://doi.org/10.1002/3527602097.
